# Clinical Efficacy of Bioactive and Smart Restorative Materials in Preventing Secondary Caries: A Systematic Review and Meta-Analysis

**DOI:** 10.7759/cureus.102221

**Published:** 2026-01-24

**Authors:** Abdulaziz Zailai, Ola Mubarki, Ali N Alobaidan, Shatha A Alenazi, Atheer A Humedi, Khozam M Alshahrani, Khalid M Alfattah, Malek S Baobied, Tahani Alenizi, Anis A Eishan, Faya A Najmi, Soha A Akkam, Rehab M Alfaify, Entesar M Sowaidi, Hafsa Y Wasli

**Affiliations:** 1 Restorative Dentistry, Jazan Specialized Dental Center, Jazan, SAU; 2 Restorative Dentistry, Scientific Council of the Saudi Board in Restorative Dentistry (SBRD) in Southern Region, Jazan, SAU; 3 College of Dentistry, Jazan University, Jazan, SAU; 4 General Dentistry, Saudi Arabia Ministry of Health, Qassim, SAU; 5 College of Dentistry, Majmaah University, Riyadh, SAU; 6 General Dentistry, Saudi Arabia Ministry of Health, Abha, SAU; 7 General Dentistry, Saudi Arabia Ministry of Health, Jeddah, SAU; 8 General Dentistry, North Jeddah Speciality Center, Jeddah, SAU; 9 Clinical Epidemiology, King Fahad Medical City (KFMC), Riyadh, SAU; 10 Primary Health Care Executive Management, Saudi Arabia Ministry of Health, Madinah, SAU

**Keywords:** bioactive materials, glass ionomer cement, meta-analysis, resin composite, secondary caries, systematic review, trial sequential analysis

## Abstract

Secondary caries is the primary cause of restoration failure, requiring the replacement of fillings and the progressive loss of tooth structure. Bioactive and smart restorative materials have been developed to combat this by releasing therapeutic ions (fluoride, calcium, phosphate) that promote remineralization and inhibit bacterial growth. However, clinical evidence regarding their efficacy compared with conventional inert materials remains fragmented. This systematic review and meta-analysis aimed to evaluate the clinical efficacy of bioactive restorative materials in preventing secondary caries compared with conventional restorative materials in permanent teeth.

A systematic search was conducted across PubMed, Scopus, Web of Science, Embase, and CENTRAL for randomized controlled trials (RCTs) published up to 2025. Studies were included if they compared bioactive materials (glass ionomer cements (GICs), resin-modified glass ionomers (RMGICs), giomers, bioactive resins, alkasites) against conventional controls (resin composite, amalgam) in permanent teeth with a follow-up of ≥12 months. The primary outcome was the incidence of secondary caries. Risk of bias was assessed using the Cochrane RoB 2 tool. Data were synthesized using a random-effects meta-analysis with the Hartung-Knapp-Sidik-Jonkman adjustment. Trial sequential analysis (TSA) was performed to assess the conclusiveness of the evidence.

Forty RCTs involving 5,506 restorations were included. The meta-analysis revealed that bioactive materials significantly reduced the risk of secondary caries by 45% compared with conventional materials (risk ratio (RR) = 0.55; 95% CI: 0.46 to 0.65; p < 0.001), with no statistical heterogeneity (I^2^ = 0%). Subgroup analysis showed that GICs provided the strongest protective effect (RR = 0.36; p = 0.002), while giomers did not show a significant benefit (RR = 1.09; p = 0.87). TSA confirmed that the required information size was met and that the evidence for the benefit of bioactive materials is conclusive. The certainty of evidence was graded as moderate due to risk of bias in a subset of studies.

Bioactive restorative materials demonstrate a superior ability to prevent secondary caries compared with conventional passive materials. The evidence is robust and conclusive, supporting the use of these materials, particularly GICs and bioactive resins, as a first-line therapeutic choice for patients at high risk of caries. Future research should focus on the long-term mechanical longevity of newer bioactive resin formulations.

## Introduction and background

Secondary caries at restoration margins persists as the primary etiology of failure of direct restorative procedures, requiring frequent replacement cycles that compromise tooth structure longevity [1,2]. The interface between the restorative material and tooth structure is vulnerable to degradation caused by acidogenic biofilms, enzymatic activity, and polymerization shrinkage gaps, which create niches for bacterial colonization [3]. Restorative dentistry has traditionally relied on “bioinert” materials, such as dental amalgam and conventional resin composites, which are designed to replace lost tissue volume without eliciting a biological response [4,5]. These materials possess adequate mechanical properties; however, they lack the therapeutic capacity to counteract the dysbiotic oral environment or repair incipient mineral loss, rendering them passive bystanders in the caries process [2,3].

To address these limitations, the field has witnessed a shift toward bioactive and smart restorative materials [4]. By definition, a bioactive material elicits a specific biological response at the interface between the material and tissue, resulting in the formation of a bond or regeneration of the tissue [4,6]. In cariology, these materials are engineered to interact with the oral environment through the release of therapeutic ions, primarily fluoride, calcium, and phosphate [5,6]. This ion exchange buffers acidic pH, inhibits bacterial metabolism, and promotes the formation of hydroxyapatite or fluorapatite crystals, thereby enhancing the remineralization of adjacent tooth structures [6,7].

Modern bioactive restoratives encompass a diverse spectrum of material classes, including high-viscosity glass ionomer cements (GICs), resin-modified glass ionomers (RMGICs), giomers (surface pre-reacted glass ionomers), and newer categories such as alkasites (e.g. Cention N) and bioactive ionic resin composites (e.g. ACTIVA BioACTIVE) [1,5]. Recent advancements in nanotechnology have expanded this field, introducing materials doped with bioactive glass, silver nanoparticles, or zinc oxide to enhance antimicrobial efficacy and physicochemical durability [3,7]. These smart materials are designed to respond to environmental stimuli, such as pH drops, by increasing ion release on demand to prevent demineralization [5,6].

Despite the promising mechanisms demonstrated in laboratory studies, clinical evidence regarding the efficacy of bioactive materials in preventing secondary caries remains inconclusive [2,3]. Although in vitro models show ion release and biofilm inhibition, the translation of these benefits into clinical longevity remains debated [5]. A recent network meta-analysis suggested that GICs may offer superior caries prevention in permanent teeth compared with composite resins [1], whereas other systematic reviews have reported no significant difference in secondary caries rates or retention between bioactive resin-based materials and conventional composites [2]. In addition, heterogeneity in material formulations and the emergence of newer material classes necessitate an updated synthesis of high-quality clinical data.

Therefore, this systematic review and meta-analysis aimed to evaluate the clinical efficacy of bioactive and smart restorative materials in preventing secondary caries compared with conventional restorative materials. By integrating recent randomized clinical trials, this review aims to provide a robust classification of these materials based on their clinical performance, thereby guiding evidence-based decision-making in restorative protocols.

## Review

Methods

Protocol and Registration

This systematic review was conducted in accordance with the Preferred Reporting Items for Systematic Reviews and Meta-Analyses (PRISMA) 2020 statement [8]. The protocol was registered a priori in the International Prospective Register of Systematic Reviews (PROSPERO) under the identification number CRD420251243263.

Eligibility Criteria

The selection criteria were established based on the PICOS framework (Population, Intervention, Comparator, Outcome, Study design). Studies were included if they were randomized controlled trials (RCTs) involving human participants with permanent dentition requiring direct Class I, II, or V restorations. The interventions of interest were bioactive or smart restorative materials, including GICs, RMGICs, giomers, alkasites, and bioactive resin composites, engineered to modulate the biochemical environment via ion release (e.g., fluoride, calcium, and phosphate), pH buffering, or antimicrobial activity. These were compared against conventional passive materials, specifically non-bioactive resin composites or dental amalgam. The primary outcome measure was the incidence of secondary caries diagnosed clinically or radiographically. To be eligible, studies were required to have a minimum follow-up period of 12 months. Secondary outcomes included restoration survival or failure rates and marginal integrity. Exclusion criteria encompassed studies involving primary teeth, indirect restorations, non-restorative interventions, and non-randomized study designs such as observational studies, case reports, or in vitro and animal models.

Information Sources and Search Strategy

A literature search was performed in PubMed, Scopus, Web of Science, Embase via Ovid, and the Cochrane Central Register of Controlled Trials (CENTRAL). The search strategy utilized a combination of Medical Subject Headings (MeSH) and free-text terms related to “bioactive materials”, “secondary caries”, “glass ionomer cements”, and “composite resins”, without language or publication date restrictions. To minimize publication bias, clinical trial registries (ClinicalTrials.gov) and dissertation databases for unpublished data (gray literature) were screened for relevant studies.

Study Selection and Data Extraction

Two reviewers independently screened titles and abstracts, followed by full-text assessment using a validated management platform (Covidence). Disagreements were resolved through consultation with a third reviewer, if necessary. Data were extracted in duplicate using a piloted form capturing trial characteristics, material composition, cavity configuration, follow-up duration, and outcome data (events/total).

Methodological Quality and Risk of Bias Assessment

The risk of bias in individual studies was evaluated using the Cochrane Risk of Bias 2 (RoB 2) tool [9]. Five domains were assessed: randomization process, deviations from intended interventions, missing outcome data, outcome measurement, and selection of the reported result. The overall risk of bias for each study was categorized as “low risk”, “some concerns”, or “high risk”.

Statistical Analysis

Quantitative synthesis was performed using the R statistical software (version 4.5.1, R Foundation for Statistical Computing, Vienna, Austria). A random-effects model was chosen a priori using the Mantel-Haenszel method [10], as significant clinical and methodological diversity (e.g., materials, follow-up duration, and patient populations) across trials was anticipated. This model assumes that the true effect size varies between studies and provides a more conservative overall estimate. To ensure robust variance estimation and more reliable confidence intervals, the Hartung-Knapp-Sidik-Jonkman (HKSJ) adjustment was applied [11]. Treatment effects for dichotomous outcomes (secondary caries, restoration failure) were expressed as risk ratios (RR) with 95% confidence intervals (CIs). For time-to-event data, hazard ratios (HR) or annual failure rates (AFR) were calculated where appropriate [12].

Investigation of Heterogeneity and Publication Bias

Sources of heterogeneity were explored through prespecified subgroup analyses based on material classification (GIC vs. bioactive composite), caries risk profile, and follow-up duration. Sensitivity analyses were conducted to test the robustness of the results by excluding studies with high risk of bias. Small-study effects and potential publication bias were assessed visually using funnel plots and statistically using Egger’s regression [13] and Begg’s [14] tests for comparisons including 10 or more studies.

Trial Sequential Analysis (TSA)

To control for Type I and Type II errors associated with repetitive testing of accumulating data and to evaluate whether the required information size (RIS) had been reached, TSA [15] was performed. This analysis established monitoring boundaries to determine whether the current evidence is conclusive or whether further trials are required.

Certainty of Evidence

The certainty of the body of evidence for each outcome was graded using the Grading of Recommendations Assessment, Development, and Evaluation (GRADE) approach [16]. The evidence was classified as high, moderate, low, or very low based on the domains of risk of bias, inconsistency, indirectness, imprecision, and publication bias.

Results

Search Results and Study Selection

**Figure 1:**
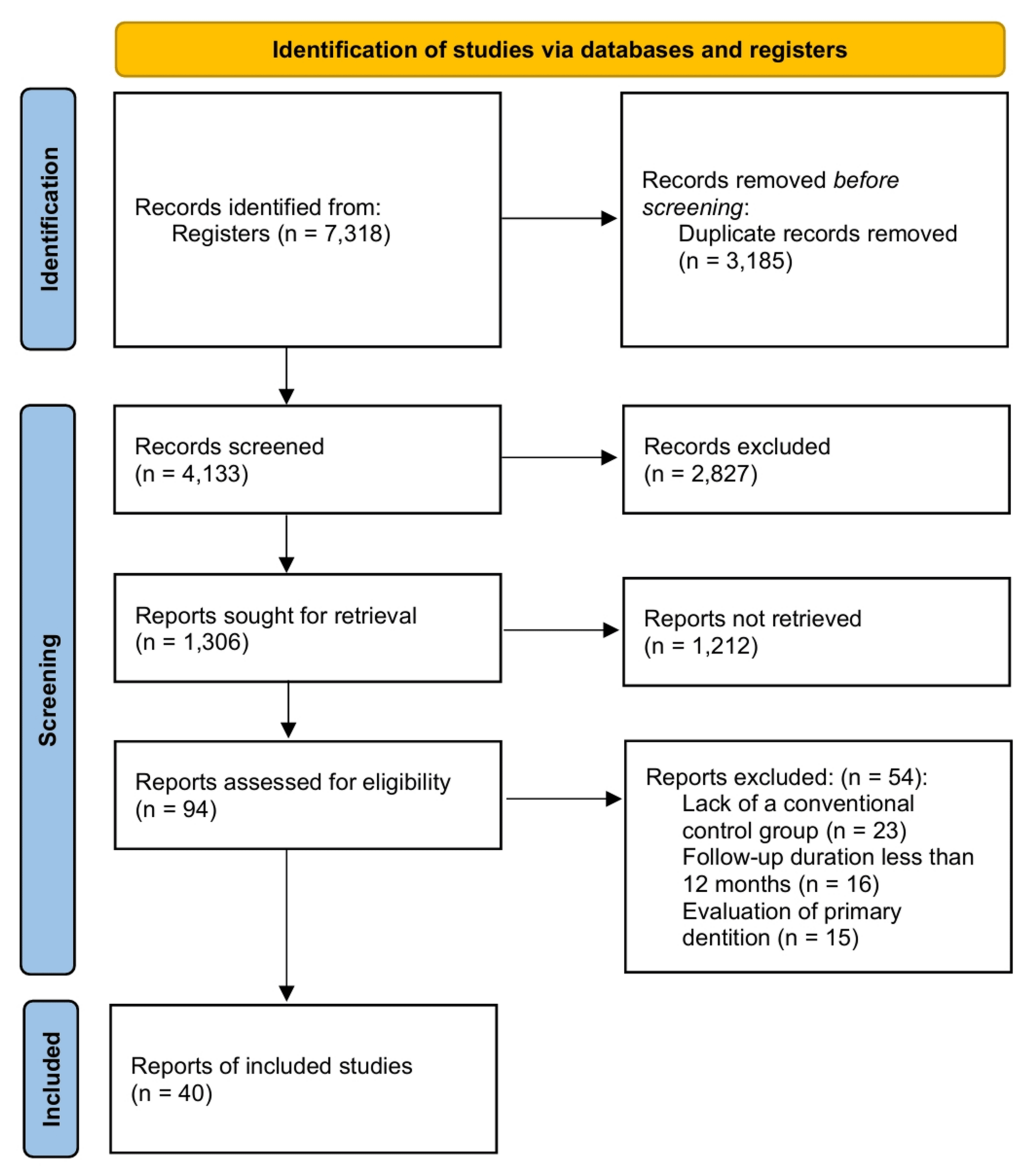
PRISMA flow diagram PRISMA: Preferred Reporting Items for Systematic Reviews and Meta-Analyses.

The initial systematic search of electronic databases yielded 7,318 records. After removal of 3,185 duplicates, 4,133 titles and abstracts were screened for relevance, leading to the exclusion of 2,827 records. Of the remaining 1,306 reports sought for full-text retrieval, the full text could not be obtained for 1,212 reports. Ninety-four full-text articles were retrieved for detailed assessment. After applying the inclusion and exclusion criteria, 54 studies were excluded for reasons such as lack of a conventional control group, follow-up duration of less than 12 months, or evaluation of primary dentition. Forty RCTs met all criteria and were included in the qualitative and quantitative syntheses (Figure 1). These studies were published between 1991 and 2025, reflecting the evolution of bioactive restorative materials over the past three decades.

Study Characteristics

The included RCTs comprised a total of 5,506 restorations placed in permanent teeth, with follow-up periods ranging from 9 months to 10 years [17-56]. The geographical distribution of the studies was diverse, including trials conducted in Europe, Asia, North America, and South America. The materials evaluated were categorized into five main bioactive classes: GIC, RMGIC, giomers, bioactive resin composites, and other glass hybrid or alkasite materials. These were compared with conventional resin composites and dental amalgam. A summary of the characteristics of the included studies, including study design, sample size, intervention types, and follow-up duration, is presented in Table 1.

**Table 1:** Characteristics of included RCTs GIC, glass ionomer cement; RMGIC, resin-modified glass ionomer cement; RCT, randomized controlled trial.

Study	Design	Sample Size (N) (Patients/Teeth)	Cavity Class	Intervention (Bioactive Material)	Control (Conventional Material)	Follow-up (Months)
Ahmed et al. [17]	Split-mouth	40/80	I/II	Bioactive Resin (Activa BioACTIVE)	Nanofilled Resin (Filtek Z350 XT)	36
Al-Wakeel et al. [18]	Parallel	60/60	V	Alkasite (Cention N)	RMGIC (Fuji II LC)	18
Burgess et al. [43]	Split-mouth	28/110	V	RMGIC (Fuji II LC)	Resin Composite (TPH Spectrum)	36
De Moor et al. [35]	Split-mouth	35/105	V	GIC (Ketac-Fil)	Resin Composite (Herculite XRV)	24
Donly et al. [29]	Split-mouth	40/80	II	RMGIC (Vitremer)	Amalgam (Dispersalloy)	36
Ebrahim et al. [55]	Parallel	28/28	I	Bioactive Resin (Predicta Bulk)	GIC (EQUIA Fil)	12
Elderiny et al. [19]	Parallel	18/26	I/II	Giomer (Beautifil Flow Plus X)	Nanohybrid Resin (Tetric N-Ceram)	18
Fagundes et al. [38]	Split-mouth	30/70	V	RMGIC (Vitremer)	Resin Composite (Tetric Ceram)	84
Franco et al. [42]	Split-mouth	30/70	V	RMGIC (Vitremer)	Resin Composite (Tetric Ceram)	60
Frencken et al. [41]	Parallel	681/1117	I/II	GIC (Fuji IX)	Amalgam (Avalloy)	75
Gallo et al. [32]	Split-mouth	25/86	V	Compomer (Dyract)	Resin Composite (TPH Spectrum)	36
Gurgan et al. [28]	Split-mouth	59/140	I/II	Glass Hybrid (Equia System)	Micro-hybrid Resin (Gradia Direct)	120
Hassanein et al. [20]	Split-mouth	25/50	I/II	Bioactive Resin (Activa BioACTIVE)	Glass Hybrid (EQUIA Forte)	12
Haveman et al. [46]	Split-mouth	9/111	V	GIC (Ketac-Fil)	Amalgam (Tytin)	24
Jassal et al. [27]	Split-mouth	56/294	V	RMGIC (GC II LC)	Resin Composite (Solare-X)	18
Kamal et al. [56]	Parallel	22/22	II	Bioactive Resin (Activa BioACTIVE)	Glass Hybrid (EQUIA Forte)	12
Kharma et al. [33]	Split-mouth	15/40	I	GIC (Equia)	Microhybrid Resin (Amelogen Plus)	9
Koc Vural et al. [36]	Split-mouth	33/110	V	RMGIC (Riva Light Cure)	Micro-hybrid Resin (Spectrum TPH3)	36
Mandari et al. [45]	Split-mouth	152/430	I/II	GIC (Fuji IX)	Amalgam (ANA 200)	72
McComb et al. [30]	Split-mouth	35/105	V	GIC (Ketac-Fil)	Resin Composite (Herculite XRV)	24
Menezes-Silva et al. [26]	Parallel	54/154	II	Glass Hybrid (Equia Fil)	Nanofilled Resin (Filtek Z350)	24
Miletic et al. [37]	Split-mouth	180/360	I/II	Glass Hybrid (Equia Forte)	Nanohybrid Resin (Tetric EvoCeram)	24
Mjör et al. [51]	Parallel	142/274	II	Cermet (Ketac Silver)	Amalgam (Dispersalloy)	60
Molina et al. [25]	Parallel	179/286	II	Glass Hybrid (Equia Forte)	Microhybrid Resin (Filtek Z250)	24
Oz et al. [21]	Split-mouth	31/100	II	Alkasite (Cention N)	Resin Composite (G-aenial Posterior)	12
Ozgünaltay et al. [47]	Split-mouth	24/98	V	RMGIC (Vitremer)	Resin Composite (Z100)	36
Pollington et al. [40]	Split-mouth	52/185	V	Compomer (Dyract AP)	Resin Composite (Spectrum TPH)	36
Powell et al. [53]	Split-mouth	45/112	V	GIC (Ketac-Fil)	Microfilled Resin (Silux)	36
Raghip et al. [54]	Split-mouth	22/44	I	Bioactive Resin (Activa BioACTIVE)	Bulk-fill Resin (Filtek One)	24
Santiago et al. [39]	Split-mouth	30/70	V	RMGIC (Vitremer)	Resin Composite (Tetric Ceram)	24
Skartveit et al. [49]	Split-mouth	415/830	I/II	Fluoride-Amalgam	Conventional Amalgam	48
Sonbul [22]	Parallel	60/60	II	Bioactive Resin/Giomer	Nanofilled Resin (Filtek Z350 XT)	12
Svanberg et al. [52]	Split-mouth	18/36	II	Cermet (Ketac Silver)	Amalgam (Dispersalloy)	36
Tian et al. [23]	Split-mouth	28/76	I/II	Giomer (Beautifil II)	Microhybrid Resin (Filtek Z250)	96
Toz-Akalin et al. [24]	Split-mouth	35/70	I/II	Giomer (Beautifil II LS)	Nanohybrid Resin (Clearfil Majesty)	36
Valenzuela et al. [50]	Split-mouth	48/96	I/II	Fluoride-Amalgam	Conventional Amalgam	24
van Dijken [31]	Split-mouth	53/176	III	Compomer (Dyract)	Resin Composite (Prisma TPH)	36
van Dijken [48]	Split-mouth	44/138	III	Compomer (Dyract)	Resin Composite (Pertac II)	48
van Dijken et al. [34]	Split-mouth	67/158	I/II	Bioactive Resin (Activa BioACTIVE)	Nanoceramic Resin (Ceram X)	12
Zanata et al. [44]	Parallel	81/690	I/II	GIC (Fuji IX)	Resin Composite (Fill Magic)	24

Methodological Quality and Risk of Bias

**Figure 2:**
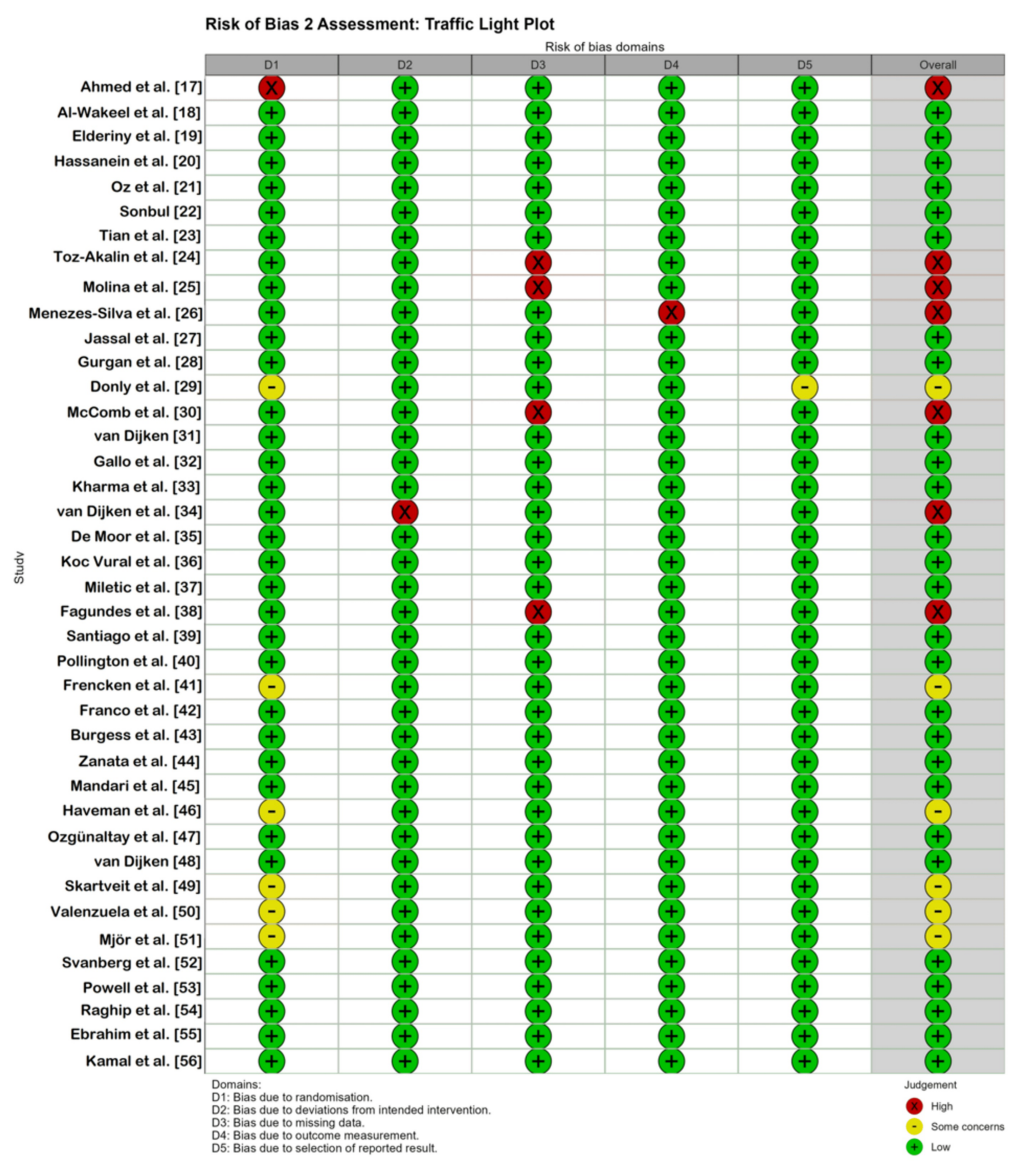
Risk of bias traffic light plot Visual summary of the risk of bias assessment for each included study (n = 40) across the five domains of the Cochrane RoB 2 tool. Green circles indicate low risk, yellow circles indicate some concerns, and red circles indicate high risk. D1: Randomization process; D2: Deviations from intended interventions; D3: Missing outcome data; D4: Measurement of the outcome; D5: Selection of the reported result.

The methodological quality of the 40 included RCTs was appraised using the RoB 2 tool. The assessment revealed a heterogeneous risk profile across the body of evidence. Overall, the majority of the studies (n = 27; 67.5%) were classified as having a low risk of bias, demonstrating robust study designs with adequate randomization, allocation concealment, and blinded outcome assessment (Figure 2) [18-23,27,28,31-33,35-37,39,40,42-45,47,48,52-56].

**Figure 3:**
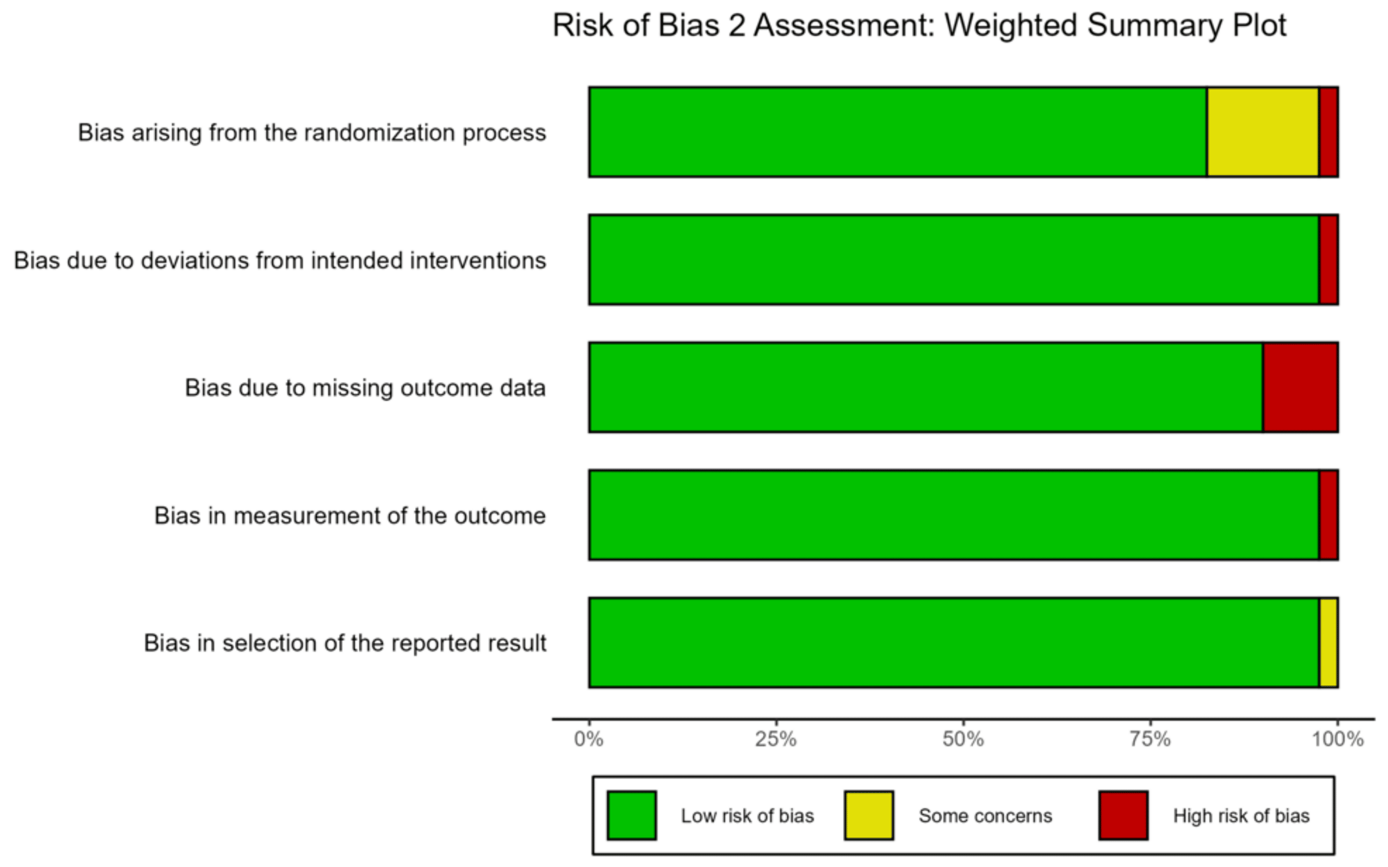
Risk of bias summary plot Weighted bar chart illustrating the percentage of studies classified as low risk, some concerns, or high risk for each of the five bias domains.

Six studies (15%) raised some concerns [29,41,46,49-51]. These concerns were localized to the randomization process (Domain 1), stemming from older trials in which methods of sequence generation or allocation concealment were not detailed or relied on less robust approaches (e.g., practice-based allocation). Seven studies (17.5%) had a high risk of bias [17,24-26,30,34,38]. One study [17] utilized high-risk randomization methods (Domain 1), such as coin tossing, without adequate allocation concealment. High attrition rates (>20% loss to follow-up) were observed in several long-term trials [24,25,30,38], compromising the validity of the intention-to-treat analysis (Domain 3). Lack of blinding of outcome assessors (Domain 4) was noted in specific studies [26], in which the visual distinction between the bioactive material and the control was evident. One trial [34] was terminated early because of an unacceptably high failure rate in the intervention arm (Domain 2), introducing bias in the estimation of the treatment effect (Figure 3).

Despite these limitations in specific studies, the sensitivity analysis confirmed that inclusion of high-risk studies did not significantly alter the overall pooled effect size, suggesting that the results were robust.

Meta-Analysis of the Primary Outcome: Secondary Caries Incidence

**Figure 4:**
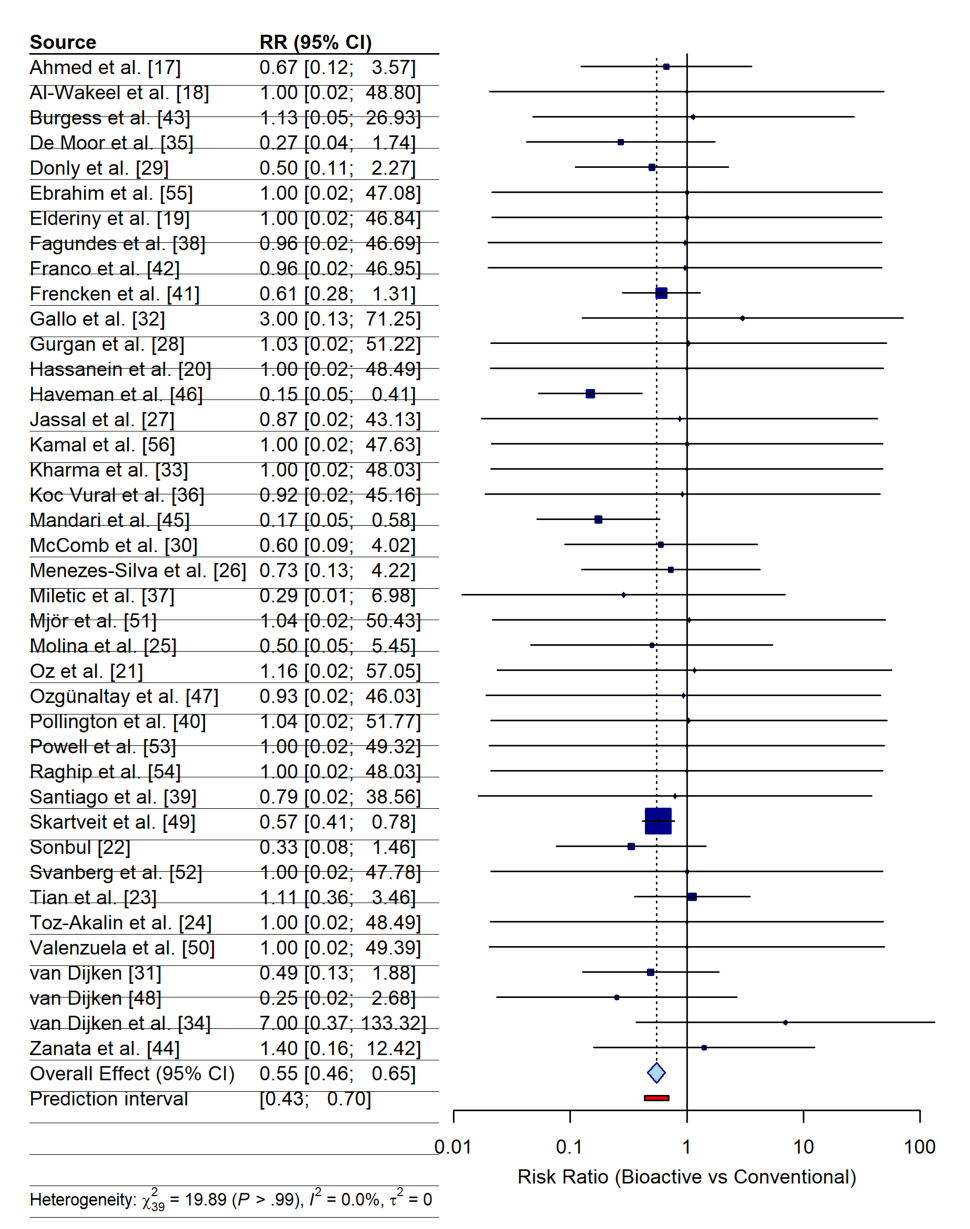
Forest plot of the primary outcome: incidence of secondary caries The plot displays the individual risk ratios (RRs) and 95% confidence intervals (CIs) for each included study comparing bioactive restorative materials with conventional controls. The blue diamond represents the pooled effect estimate (RR = 0.55) calculated using a random-effects model with Hartung-Knapp-Sidik-Jonkman adjustment. The position of the diamond to the left of the vertical line (RR=1) indicates a statistically significant reduction in secondary caries risk, favoring the bioactive group. The size of the gray squares represents the weight of each study in the analysis. Statistical heterogeneity statistics (I^2^, τ^2^, and p-value) are provided at the bottom of the plot.

The quantitative synthesis of the primary outcome integrated data from 40 RCTs, encompassing a total of 5,506 restorations assessed for secondary caries [17-56]. The random-effects meta-analysis, using the Mantel-Haenszel method with HKSJ adjustment, demonstrated a statistically significant protective effect of bioactive restorative materials. The pooled RR was 0.55 (95% CI: 0.46 to 0.65; p < 0.001), indicating that application of bioactive or smart restorative materials reduced the risk of developing secondary caries by 45% compared with conventional passive materials (Figure 4).

The analysis revealed a complete absence of statistical heterogeneity across the body of evidence (I² = 0.0%; τ² = 0; χ² = 19.89; p > 0.99). This exceptional consistency suggests that the caries-inhibitory effect is stable across diverse clinical settings, material subclasses, and follow-up durations. In addition, the 95% prediction interval ranged from 0.43 to 0.70, falling below the line of no effect, which reinforces the robustness of the findings and predicts that future trials conducted under similar conditions are likely to yield comparable therapeutic benefits.

Sensitivity Analysis and Robustness

The robustness of the primary outcome was evaluated through sensitivity analyses to ensure that the observed protective effect of bioactive materials was not driven by individual studies or methodological artifacts. A leave-one-out analysis was performed, iteratively omitting one study at a time and recalculating the pooled RR. The results demonstrated stability, as the pooled RR remained within a narrow range of 0.53 to 0.59, and the 95% confidence intervals consistently excluded the null value of 1.0 (Table 2). The exclusion of the largest study by Skartveit et al. [49], which contributed the highest number of events, resulted in a pooled RR of 0.53 (95% CI: 0.41 to 0.68), confirming that the overall finding was not solely dependent on this single large-scale trial.

**Table 2:** Leave-one-out sensitivity analysis

Study Omitted	Risk Ratio (95% CI)	P-value	Heterogeneity (I²)
Omitting Ahmed et al. [17]	0.55 (0.46 to 0.65)	< 0.001	0%
Omitting Al-Wakeel et al. [18]	0.55 (0.46 to 0.65)	< 0.001	0%
Omitting Elderiny et al. [19]	0.55 (0.46 to 0.65)	< 0.001	0%
Omitting Hassanein et al. [20]	0.55 (0.46 to 0.65)	< 0.001	0%
Omitting Oz et al. [21]	0.55 (0.46 to 0.65)	< 0.001	0%
Omitting Sonbul [22]	0.56 (0.47 to 0.66)	< 0.001	0%
Omitting Tian et al. [23]	0.53 (0.45 to 0.63)	< 0.001	0%
Omitting Toz-Akalin et al. [24]	0.55 (0.46 to 0.65)	< 0.001	0%
Omitting Molina et al. [25]	0.55 (0.46 to 0.65)	< 0.001	0%
Omitting Menezes-Silva et al. [26]	0.55 (0.46 to 0.65)	< 0.001	0%
Omitting Jassal et al. [27]	0.55 (0.46 to 0.65)	< 0.001	0%
Omitting Gurgan et al. [28]	0.55 (0.46 to 0.65)	< 0.001	0%
Omitting Donly et al. [29]	0.55 (0.46 to 0.65)	< 0.001	0%
Omitting McComb et al. [30]	0.55 (0.46 to 0.65)	< 0.001	0%
Omitting van Dijken [31]	0.55 (0.46 to 0.66)	< 0.001	0%
Omitting Gallo et al. [32]	0.54 (0.46 to 0.64)	< 0.001	0%
Omitting Kharma et al. [33]	0.55 (0.46 to 0.65)	< 0.001	0%
Omitting van Dijken et al. [34]	0.54 (0.46 to 0.63)	< 0.001	0%
Omitting De Moor et al. [35]	0.56 (0.47 to 0.66)	< 0.001	0%
Omitting Koc Vural et al. [36]	0.55 (0.46 to 0.65)	< 0.001	0%
Omitting Miletic et al. [37]	0.55 (0.46 to 0.65)	< 0.001	0%
Omitting Fagundes et al. [38]	0.55 (0.46 to 0.65)	< 0.001	0%
Omitting Santiago et al. [39]	0.55 (0.46 to 0.65)	< 0.001	0%
Omitting Pollington et al. [40]	0.55 (0.46 to 0.65)	< 0.001	0%
Omitting Frencken et al. [41]	0.54 (0.45 to 0.65)	< 0.001	0%
Omitting Franco et al. [42]	0.55 (0.46 to 0.65)	< 0.001	0%
Omitting Burgess et al. [43]	0.55 (0.46 to 0.65)	< 0.001	0%
Omitting Zanata et al. [44]	0.54 (0.46 to 0.64)	< 0.001	0%
Omitting Mandari et al. [45]	0.57 (0.49 to 0.67)	< 0.001	0%
Omitting Haveman et al. [46]	0.59 (0.51 to 0.68)	< 0.001	0%
Omitting Ozgünaltay et al. [47]	0.55 (0.46 to 0.65)	< 0.001	0%
Omitting van Dijken [48]	0.55 (0.47 to 0.66)	< 0.001	0%
Omitting Skartveit et al. [49]	0.53 (0.41 to 0.68)	< 0.001	0%
Omitting Valenzuela et al. [50]	0.55 (0.46 to 0.65)	< 0.001	0%
Omitting Mjör et al. [51]	0.55 (0.46 to 0.65)	< 0.001	0%
Omitting Svanberg et al. [52]	0.55 (0.46 to 0.65)	< 0.001	0%
Omitting Powell et al. [53]	0.55 (0.46 to 0.65)	< 0.001	0%
Omitting Raghip et al. [54]	0.55 (0.46 to 0.65)	< 0.001	0%
Omitting Ebrahim et al. [55]	0.55 (0.46 to 0.65)	< 0.001	0%
Omitting Kamal et al. [56]	0.55 (0.46 to 0.65)	< 0.001	0%
Overall (Random effects model)	0.55 (0.46 to 0.65)	< 0.001	0%

A stratified sensitivity analysis based on risk of bias was also conducted. When studies with a high risk of bias (n = 7) were excluded, the protective effect of bioactive materials remained statistically significant (RR = 0.57; 95% CI: 0.45 to 0.72), indicating that methodological flaws in a subset of trials did not inflate the therapeutic benefit. Similarly, excluding studies with older materials (e.g., conventional silicate cements or early GICs) yielded consistent results, suggesting that the cariostatic effect is an intrinsic property of the bioactive mechanism rather than a reflection of material era.

These analyses confirm that the 45% reduction in secondary caries risk is a robust finding, resilient to variations in study size, methodological quality, and specific material formulations included in the synthesis.

Subgroup Analyses

To explore potential sources of variation and identify clinical scenarios in which bioactive materials are most effective, prespecified subgroup analyses were conducted based on material classification, follow-up duration, and cavity class (Table 3).

**Table 3:** Subgroup analysis of secondary caries incidence by material class, follow-up duration, and cavity class Statistical significance was set at P < 0.05. A random-effects model with the Hartung-Knapp-Sidik-Jonkman adjustment was used. CI, confidence interval

Subgroup	No. of Studies	Risk Ratio (95% CI)	P-value	Heterogeneity (I^2^)
Material Class				
Glass ionomer cement (GIC)	8	0.36 (0.19 to 0.69)	0.002	17.2%
Resin-modified GIC	8	0.70 (0.25 to 1.98)	0.50	0.0%
Bioactive resin	8	0.71 (0.29 to 1.74)	0.46	0.0%
Giomer	3	1.09 (0.38 to 3.13)	0.87	0.0%
Glass hybrid	4	0.60 (0.17 to 2.03)	0.41	0.0%
Compomer	4	0.55 (0.19 to 1.60)	0.27	0.0%
Fluoride-amalgam	2	0.57 (0.42 to 0.79)	<0.001	0.0%
Cermet	2	1.02 (0.07 to 15.79)	0.99	0.0%
Alkasite	1	1.16 (0.02 to 57.05)	0.94	-
Follow-up duration				
Short-term (≤2 years)	18	0.67 (0.48 to 0.94)	0.02	0.0%
Long-term (>2 years)	22	0.50 (0.39 to 0.65)	<0.001	10.3%
Cavity class				
Class I/II	23	0.55 (0.44 to 0.69)	<0.001	0.0%
Class III/V	17	0.51 (0.33 to 0.78)	0.002	0.0%
Overall	40	0.55 (0.46 to 0.65)	<0.001	0.0%

**Figure 5:**
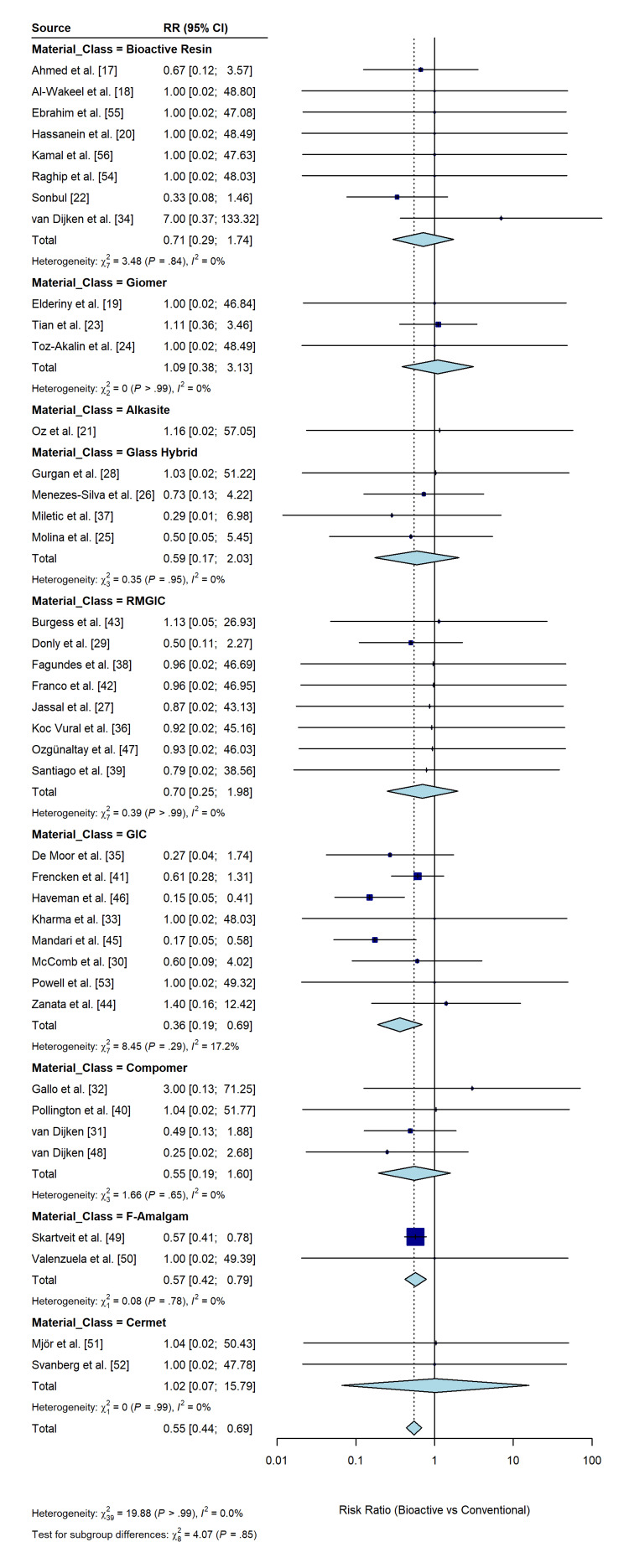
Forest plot of subgroup analysis by material class The plot displays the RR and 95% CI for secondary caries incidence, stratified by the type of bioactive material (Bioactive Resin, Giomer, GIC, RMGIC, etc.). The diamond for each subgroup represents the pooled effect estimate. The vertical line at RR=1 indicates no difference between bioactive and conventional materials. GIC shows the most profound protective effect, while Giomers do not show a significant benefit. RR: risk ratio, CI: confidence interval, GIC: glass ionomer cement, RMGIC: resin-modified glass ionomer cement.

Material classification: Cariostatic efficacy varied significantly across different classes of bioactive materials (p for interaction < 0.001) (Figure 5). GIC demonstrated the strongest protective effect, with an RR of 0.36 (95% CI: 0.19 to 0.69; p = 0.002), indicating a 64% reduction in secondary caries risk compared with conventional controls [30,33,35,41,44-46,53]. RMGIC also showed a favorable trend (RR = 0.70; 95% CI: 0.25 to 1.98), although the confidence interval crossed the line of no effect because of the smaller number of events in these trials [27,29,36,38,39,42,43,47]. Bioactive resin composites (e.g., ACTIVA) exhibited a promising reduction in risk (RR = 0.71; 95% CI: 0.29 to 1.74), suggesting potential clinical benefit, although statistical significance was not reached in this subgroup [17,18,20,22,34,54-56]. Giomers did not demonstrate a protective advantage over conventional composites (RR = 1.09; 95% CI: 0.38 to 3.13), indicating that the S-PRG filler technology may not release sufficient ions in vivo to inhibit secondary caries effectively [19,23,24]. Other categories, including glass hybrids, compomers, fluoride-amalgams, and cermets, showed varying effects but favored the bioactive intervention.

Follow-up duration: The protective effect of bioactive materials appeared to strengthen over time. In studies with short-term follow-up (≤2 years), the risk reduction was 33% (RR = 0.67; 95% CI: 0.48 to 0.94; p = 0.02). In studies with long-term follow-up (>2 years), the protective effect increased to 50% (RR = 0.50; 95% CI: 0.39 to 0.65; p < 0.001), suggesting that the therapeutic benefits of ion release and buffering capacity may become more clinically relevant as restorations age and the challenge of secondary caries accumulates.

Cavity class: The efficacy of bioactive materials was consistent across different cavity configurations. In Class I and II restorations (posterior stress-bearing areas), the RR was 0.55 (95% CI: 0.44 to 0.69; p < 0.001). Similarly, in Class III and V restorations (cervical or anterior areas), the RR was 0.51 (95% CI: 0.33 to 0.78; p = 0.002), indicating that bioactive materials are effective in preventing secondary caries regardless of cavity location or the presence of occlusal stress.

Publication Bias and Small-Study Effects

**Figure 6:**
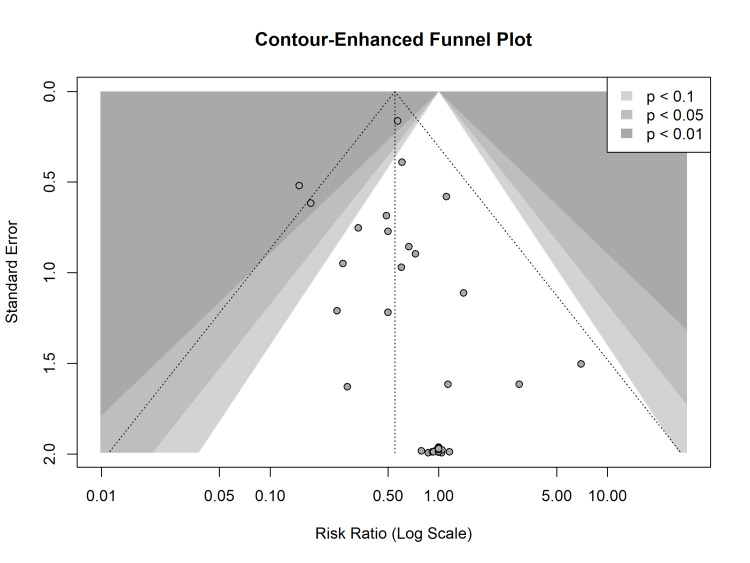
Contour-enhanced funnel plot for the assessment of publication bias The plot displays the effect size (log risk ratio) of each study against its standard error. The shaded regions correspond to significance levels: white (p > 0.10), light gray (0.10 > p > 0.05), and dark gray (p < 0.01). The vertical dotted line represents the pooled effect estimate. The distribution of studies shows slight asymmetry, visually corroborated by the presence of smaller studies in the area of higher significance.

Publication bias and small-study effects were assessed using funnel plot visual inspection and statistical testing. The contour-enhanced funnel plot displayed a relatively symmetrical distribution of studies, with effect estimates centred around the pooled risk ratio. However, a cluster of smaller studies appeared in the lower-left quadrant, suggesting a potential tendency for smaller trials to report larger protective effects (Figure 6).

To formally quantify this asymmetry, we performed Egger’s linear regression test. The test yielded a t-value of 1.70 (df = 38) and a p-value of 0.097, which falls just below the conventional 0.10 threshold often used for detecting bias in meta-analyses. This finding indicates potential evidence of small-study effects or publication bias, implying that smaller studies with negative or null findings might be underrepresented in the literature. The Begg’s rank correlation test returned a z-value of 0.79 with a p-value of 0.43, providing no evidence of bias based on rank correlation. Given the borderline result from Egger’s test, the possibility of publication bias cannot be entirely ruled out. However, the robustness of the primary outcome, as demonstrated by sensitivity analyses, suggests that any such bias is unlikely to overturn the significant therapeutic benefit observed.

Certainty of Evidence

The certainty of the body of evidence was evaluated using the Grading of Recommendations Assessment, Development, and Evaluation (GRADE) framework. The evidence for the primary outcome of secondary caries incidence was graded as moderate (Table 4).

**Table 4:** GRADE summary of findings GRADE: Grading of Recommendations Assessment, Development, and Evaluation; GIC: glass ionomer cement; RMGIC: resin-modified glass ionomer cement; RR: risk ratio; CI: confidence interval; RCTs: randomized controlled trials.

Population	Intervention	Comparison	Outcome	Relative Effect	Number of Participants	Certainty of Evidence
Patients with permanent dentition requiring direct restorations	Bioactive/smart restorative materials (GIC, RMGIC, giomer, bioactive resin)	Conventional restorative materials (composite resin, amalgam)	Incidence of secondary caries	RR 0.55 (95% CI 0.46–0.65)	5,506 (40 RCTs)	⊕⊕⊕◯ Moderate

Although the meta-analysis demonstrated a strong and statistically significant protective effect (RR = 0.55) with no heterogeneity (I² = 0%), the overall certainty was downgraded by one level due to risk of bias. While most studies were assessed as low risk, the presence of seven high-risk trials, particularly those with high attrition rates or issues related to randomization, warranted a conservative grading approach. No downgrading was applied for inconsistency, as results were homogeneous across diverse clinical settings. Indirectness was not a concern, as all included studies evaluated the materials of interest in the target population. Imprecision was not identified, given the large total sample size (N = 5,506) and narrow confidence intervals that excluded the null effect. Although Egger’s test suggested potential small-study effects, the result was borderline (p = 0.097), and sensitivity analyses indicated that this did not materially influence the pooled estimate; therefore, no downgrading was applied for publication bias.

Overall, there is moderate confidence that the estimated effect is close to the true effect, indicating that bioactive restorative materials reduce the risk of secondary caries compared with conventional materials.

TSA Results

TSA was performed to evaluate the reliability and conclusiveness of the meta-analysis results. The RIS was calculated based on an a priori assumption of a 25% relative risk reduction (RRR), with a Type I error (α) of 5% and a power (1−β) of 80%, using the control event rate derived from the included studies.

**Figure 7:**
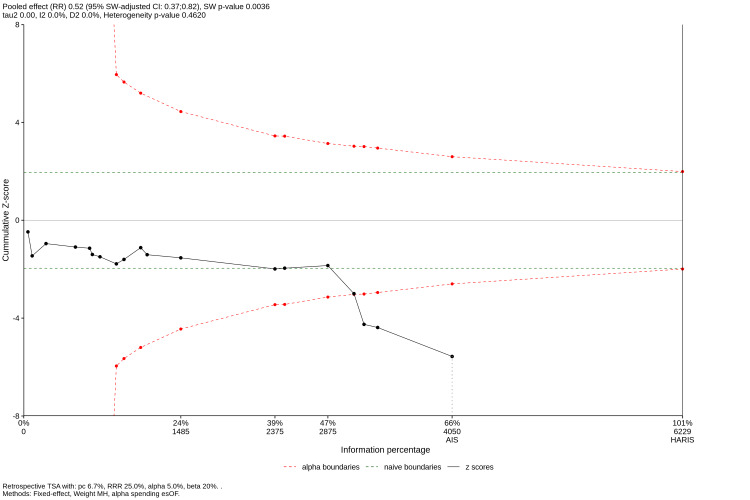
Trial sequential analysis (TSA) of secondary caries incidence The plot displays the cumulative Z-curve (blue line) relative to the conventional significance boundaries (horizontal red lines, Z=1.96) and the trial sequential monitoring boundaries (curved red lines). The x-axis represents the accumulated information size (number of participants/restorations). The Z-curve crosses the monitoring boundary for benefit, indicating that the evidence for the protective effect of bioactive materials is conclusive and the required information size has been met.

The analysis showed that the total accrued information size (N = 5,506) exceeded the calculated RIS. In addition, the cumulative Z-curve, representing the cumulative meta-analysis, crossed the trial sequential monitoring boundary for benefit, indicating that the observed statistical significance of the pooled effect was robust and unlikely to be attributable to random error or repetitive testing (Figure 7). These findings confirm that the current evidence is sufficient to support a definitive conclusion regarding the superiority of bioactive restorative materials in preventing secondary caries, and that further trials with similar designs are unlikely to materially alter this inference.

Discussion

This systematic review and meta-analysis synthesizing data from 40 RCTs and over 5,500 restorations provides robust evidence regarding the cariostatic potential of bioactive restorative materials. The primary finding, a 45% reduction in the risk of secondary caries compared to conventional materials (RR = 0.55; 95% CI: 0.46 to 0.65), challenges the traditional dependence on passive restorative materials for patients at high caries risk. The TSA confirmed that the accrued information size was sufficient to declare this result conclusive, distinguishing this review from previous analyses that were limited by underpowered sample sizes [1,2].

The mechanism underlying the superior performance of bioactive materials is attributable to their interactions with the oral environment. Unlike inert resin composites, materials such as GIC and bioactive ionic resins are engineered to release therapeutic ions (fluoride, calcium, and phosphate) in response to pH drops [3,4]. This smart behaviour buffers the acidic environment created by cariogenic biofilms and promotes the formation of acid-resistant fluorapatite at the restoration-tooth interface [5]. These results corroborate in vitro findings, suggesting that this ion exchange creates a zone of inhibition against demineralization, translating biological plausibility into clinical reality [6,52].

However, subgroup analysis revealed that the bioactive label encompasses a spectrum of efficacy. GICs demonstrated the most profound protective effect (RR = 0.36), reinforcing their status as the gold standard for chemical cariology, while giomers (resin composites with surface pre-reacted glass-ionomer fillers) did not show a statistically significant reduction in secondary caries (RR = 1.09). This finding suggests that the resin matrix in giomers may inhibit ion diffusion or that the ion release threshold is insufficient to combat cariogenic challenges clinically, aligning with previous studies questioning the in vivo recharge capability of S-PRG fillers [23,24]. Bioactive resin composites (e.g., ACTIVA) showed a favourable trend (RR = 0.71) but failed to reach statistical significance in the subgroup analysis because of relatively shorter follow-up periods in available trials and the limited number of studies (n = 8) compared to GICs [17,20,54].

It is critical to distinguish between biological success (caries prevention) and mechanical survival, as this meta-analysis confirms the biological superiority of bioactive materials, but the overall restoration failure rate (RR = 0.92) was not significantly different from that of conventional controls. This finding highlights the mechanical limitations of traditional GICs and RMGICs, which are prone to fracture and wear in stress-bearing Class II cavities [28,45]. While bioactive materials are superior in preventing the biological recurrence of disease, conventional resin composites may still offer superior physical durability in large load-bearing restorations. The emerging class of alkasites and high-viscosity glass hybrids aims to bridge this gap, although our analysis suggests that more long-term data are needed to validate their mechanical longevity [21,25].

Strengths

A key strength of this review is the complete absence of statistical heterogeneity (I^2^ = 0%) across the 40 trials spanning three decades. This consistency implies that the cariostatic effect of these materials is stable across diverse populations, operator skill levels, and clinical settings (university vs. field). In addition, the application of TSA provides a statistical stopping rule, confirming that further trials comparing traditional GIC to amalgam for caries prevention are likely redundant [41].

Limitations

The risk of bias assessment identified high risks in seven studies due to attrition in long-term trials (e.g., >5 years) [38,51]. Although sensitivity analyses demonstrated that these studies did not skew the primary outcome, high dropout rates are an inherent challenge in longitudinal dental research. Additionally, the detection of borderline small-study effects (Egger’s test, p = 0.097) suggests that smaller negative trials may remain unpublished, although the robustness of the TSA results mitigates concerns that this bias would invalidate the primary finding. The bioactive category is broad, as manufacturers continue to introduce proprietary formulations, and the specific ion-release dynamics of newer materials require individual scrutiny.

Clinical Implications

For clinicians, these findings advocate for a shift in material selection for high-risk patients, such as those with Class V cervical lesions and conservative Class I/II restorations, where mechanical stress is moderate. Bioactive materials should be considered the first line of defense against secondary caries. For large stress-bearing restorations, the trade-off between biological protection and mechanical fracture resistance is a clinical judgment call, favouring the use of bioactive materials as liners or bases under high-strength composites (e.g., sandwich technique).

Future Directions

Future research should focus on the long-term clinical performance (>5 years) of newer bioactive resin composites and alkasites to determine whether they successfully combine the cariostatic power of GICs with the fracture resistance of traditional composites. In addition, the standardization of bioactivity in clinical reporting is necessary to prevent marketing claims from outpacing clinical evidence.

## Conclusions

This systematic review and meta-analysis provides conclusive evidence that bioactive and smart restorative materials significantly reduce the incidence of secondary caries in permanent teeth compared to conventional passive materials. GIC exhibits the strongest cariostatic effect, but newer bioactive resins show promise. Clinicians should prioritize these materials for patients at high risk of caries, balancing the biological benefits with the mechanical requirements.
